# SEARCH ANNOUNCEMENT: Editors-in-Chief: *Advances in Nutrition and The Journal of Nutrition*

**DOI:** 10.1093/jn/nxac196

**Published:** 2022-08-30

The American Society for Nutrition (ASN) announces the search for Editors-in-Chief for two of its premier journals: ***Advances in Nutrition****(AN) and****The Journal of Nutrition****(JN)*.

These are exciting opportunities for talented, motivated, and innovative leaders to contribute to the advancement of nutrition science. ASN encourages nominations and self-nominations from interested and qualified candidates around the world and from diverse backgrounds.

Editors-in-Chief are expected to be officially appointed in spring 2023. The five-year terms begin as follows: August 1, 2023 (*AN* Editor-in-Chief) and January 1, 2024 (*JN* Editor-in Chief).

Applicants should have a broad perspective of the nutrition science field and astute knowledge of emerging areas of research and practice. Recognized leadership in rigorous nutrition science research and scholarly publication is essential.

Applications must be submitted by **October 28, 2022**. Please include:

- Letter of interest and journal to which you are applying (AN or JN)
- Curriculum vitae that includes editorial experience and a robust publication record with scientific journals in nutrition fields

**Table:** 

Advances in Nutrition	The Journal of Nutrition
2021 Impact Factor: 11.567	2021 Impact Factor: 4.687
#2 of 90 journals in Nutrition & Dietetics	Prestigious 100-year history
Literature reviews focused on key findings and the most recent research in all areas of interest to nutrition and biomedical researchers	Original research, reviews, and other content related to human, animal, population, cellular, and molecular nutrition

 

Details and job descriptions can be found at https://nutrition.org/publications/editor-search/

ASN has partnered with scholarly publishing experts KGL Consulting to conduct the search for incoming Editors-in-Chief. Questions and applications should be directed to Ms. Lisa Marshall, Senior Consulting Associate, KGL Consulting (lisa.marshall@kwglobal.com).

## Notes

Application Deadline: October 28, 2022

Address correspondence to GBT (e-mail: gtwillman@nutrition.org).

